# The role of ERBB4 mutations in the prognosis of advanced non-small cell lung cancer treated with immune checkpoint inhibitors

**DOI:** 10.1186/s10020-021-00387-z

**Published:** 2021-10-07

**Authors:** Xilin Hu, Hanlin Xu, Qianwen Xue, Ruran Wen, Wenjie Jiao, Kaihua Tian

**Affiliations:** 1 Department of Thoracic Surgery, The Affiliated Hospital of Qingdao University, Qingdao, 266000 Shandong China; 2 Qingdao Maternal & Child Health and Family Planning Service Center, Qingdao, 266000 Shandong China

**Keywords:** NSCLC, ERBB4, TP53, Immune checkpoint inhibitors, Co-occurencing mutations, Nomogram

## Abstract

**Background:**

Immune checkpoint inhibitors (ICIs) have witnessed the achievements of convincing clinical benefits that feature the significantly prolonged overall survival (OS) of patients suffering from advanced non-small cell lung cancer (NSCLC), according to reports recently. Sensitivity to immunotherapy is related to several biomarkers, such as PD-L1 expression, TMB level, MSI-H and MMR. However, a further investigation into the novel biomarkers of the prognosis on ICIs treatment is required. In addition, there is an urgent demand for the establishment of a systematic hazard model to assess the efficacy of ICIs therapy for advanced NSCLC patients.

**Methods:**

In this study, the gene mutation and clinical data of NSCLC patients was obtained from the TCGA database, followed by the analysis of the detailed clinical information and mutational data relating to two advanced NSCLC cohorts receiving the ICIs treatment from the cBioPortal of Cancer Genomics. The Kaplan–Meier plot method was used to perform survival analyses, while selected variables were adopted to develop a systematic nomogram. The prognostic significance of ERBB4 in pan-cancer was analyzed by another cohort from the cBioPortal of Cancer Genomics.

**Results:**

The mutation frequencies of TP53 and ERBB4 were 54% and 8% in NSCLC, respectively. The mutual exclusive analysis in cBioPortal has indicated that ERBB4 does show co-occurencing mutations with TP53. Patients with ERBB4 mutations were confirmed to have better prognosis for ICIs treatment, compared to those seeing ERBB4 wild type (PFS: exact *p* = 0.017; OS: exact *p* < 0.01) and only TP53 mutations (OS: *p* = 0.021). The mutation status of ERBB4 and TP53 was tightly linked to DCB of ICIs treatment, PD-L1 expression, TMB value, and TIICs. Finally, a novel nomogram was built to evaluate the efficacy of ICIs therapy.

**Conclusion:**

ERBB4 mutations could serve as a predictive biomarker for the prognosis of ICIs treatment. The systematic nomogram was proven to have the great potential for evaluating the efficacy of ICIs therapy for advanced NSCLC patients.

## Introduction

Among various cancers, lung cancer accounts for the highest incidence and is the leading cause of cancer-related death worldwide (Bray et al. 2018). Lung cancer can be classified into two pathological forms, i.e., non-small cell lung cancer (NSCLC) and small cell lung carcinoma (SCLC). The former accounts for 85% of lung cancer with a 5-year survival rate of advanced NSCLC patients ranging from 5 to 10% (Siegel et al. 2020). The study of ICIs to the end, including atezolizumab, pembrolizumab, and nivolumab, has achieved convincing clinical benefits, significantly prolonging the OS of patients (Mok et al. 2019; Horn et al. 2017; Leighl et al. 2019). However, the objective response rate of ICIs therapy is only nearly 17%, illustrating that the majority have failed to get benefits from ICIs (Park et al. 2018; Brahmer et al. 2012). Consequently, more effort should be made in the identification of appropriate patients who may respond to ICIs therapy.

The relationship between sensitivity to immunotherapy and several biomarkers has been discovered recently, such as programmed death-ligand 1 (PD-L1) expression (Mok et al. 2019; Ready et al. 2019; Ott et al. 2019), tumor mutational burden (TMB) (Ready et al. 2019; Ott et al. 2019), microsatellite instability-high (MSI-H) (Li et al. 2020), and mismatch repair (MMR) (Mandal et al. 2019). Among them, only the expression level of PD-L1 is insufficient regarding the prediction of the prognosis of immunotherapy in NSCLC (Reck et al. 2016; Taube et al. 2014), while TMB has been regarded as an appropriate predictive biomarker for immunotherapy efficacy in various cancers, such as NSCLC (Goodman et al. 2017; Gubin et al. 2015; McGranahan et al. 2016). However, it is difficult for advanced NSCLC patients to assess the level of TMB, given the high cost of next generation sequencing (NGS) and the requirement of adequate pathologic tissue. Moreover, the dynamic changes of TMB can be caused by cancer progression and treatment (Cyriac and Gandhi 2018). Tumor cell proliferation is activated through the specific somatic mutation in driver genes. Patients with the mutation of EGFR or ALK might not get clinical benefits from ICIs therapy. Therefore, it is an urgent demand to construct a hazard model, involving the biomarkers aforementioned and the genetic alterations for the prognosis of immunotherapy.

Tyrosine kinase receptors of the ERBB family include four members: HER-1/ERBB1/EGFR, HER-2/ERBB2, HER-3/ERBB3, and HER-4/ERBB4 (Bouchez et al. 2020; Li et al. 2016). The previous research has indicated that the ERBB receptor family is closely associated with cell proliferation and oncogenic events (Hyman et al. 2018). As an exception, ERBB4 is the only one with growth inhibiting and differentiation stimulating abilities, the expression of which has been proven to be downregulated in different types of aggressive tumors (Muraoka-Cook et al. 2008; Naresh et al. 2006). The functional characterization of its nine mutations has disclosed four types of activating mutations (K935I, D931Y, Y285C, and D595V) with the levels of increased basal and ligand-induced ERBB4 phosphorylation (Kurppa et al. 2016). Moreover, certain ERBB4 polymorphisms (SNPs rs6742399, rs6740117, and rs6747637) are firmly linked to a higher risk of lung cancer, suggesting that ERBB4 mutation might predispose to the development of lung cancer (Zhang et al. 2017). The similarity between the sequences of EGFR and ERBB4 kinase domains is 79% (Olayioye et al. 2000). It is well-known that EGFR-TKIs are the first-line treatments for advanced NSCLC patients with EGFR mutations. Previous studies have reported that EGFR mutations can be regarded as biomarkers of resistance to ICIs (Garassino et al. 2018; Lee et al. 2017). According to the latest research, they are mainly enriched in NSCLC patients with high PD-L1 expression, compared to those with other ERBB family numbers (Wang et al. 2021a, b). Several reports have suggested that patients with ERBB4 mutations may respond to ICIs treatment in esophageal cancer and cervical cancer (Ngoi et al. 2018; Yan et al. 2019). As a result, ERBB4 mutations may be a novel biomarker for lung cancer immunotherapy.

The study of two immunotherapy cohorts with clinical and mutational data (Rizvi et al. 2018; Samstein et al. 2019) has been conducted to clarify the relationship between ERBB4 mutations and the prognosis of NSCLC patients receiving ICIs treatment. The Cancer Genome Atlas (TCGA)-NSCLC cohort has been collected to explore the role of ERBB4 mutations in the tumor-infiltrating immune cells and TMB level. At the end, a systematic nomogram predicting the prognosis of NSCLC patients with ICIs therapy has been established based on the clinicopathological information and mutational data.

## Methods

### Study design and data download

Two immunotherapy cohorts with clinical and mutational data were collected from the cBioPortal. The first cohort [MSKCC, J Clin Oncol 2018] was comprised of 240 advanced NSCLC patients with mutational data who received ICI therapy (pembrolizumab/pembrolizumab + ipilimumab), and 86 of them accepted PD-L1 expression assessment (Rizvi et al. 2018). The efficacy of the immunotherapy was assessed by the percentages of durable clinical benefit (DCB) according to RECIST version 1.1 (Kuhl et al. 2019). PFS (Progress free survival) was assessed as the time period from the date of ICIs treatment to the date of progression or death in this cohort. The other cohort [TMB and Immunotherapy (MSKCC, Nat Genet 2019)] consisted of 350 advanced NSCLC patients receiving FDA-approved ICIs therapy (Samstein et al. 2019). OS was assessed as the date of death or last follow-up. The assessment of TMB was reported in both clinical trials. In addition, the analysis of the somatic mutation and survival data from the Cancer Genome Atlas (TCGA)-LUAD and -LUSC cohort were carried out.

### Development of nomogram

The prognosis univariates were analyzed via the Kaplan–Meier method, and the log rank tests were used to detect significant differences. A nomogram was formulated based on the results of univariate analyses with the “rms” package of R software version 3.1.2. The accuracy and discriminative value of the nomogram was estimated by concordance index (C-index) and Calibration plot.

### Bioinformatic analysis

The gene mutation, expression, and clinical data were downloaded from TCGA portal. LUAD and LUSC patients were divided into two groups according to the mutation status of ERBB4, respectively. All base substitutions and indels in the coding region of targeted genes were counted instead of silent mutations failing to contribute to an amino acid change. To calculate the TMB score of each sample, the total number of mutations counted was divided by the exome size (that 38 Mb was used as the estimation of the exome size). The gene set enrichment analysis (GSEA) was performed through Broad Institute GSEA software 4.1. The different degree of whole genes was obtained and ranked based on the comparison between ERBB4 mutation group and normal group, which was followed by the GSEA analysis. Permutations were set to 1000 in a bid to obtain a normalized enrichment score (NES). A normal p-value < 0.05 was considered significantly enriched. CIBERSORT (Newman et al. 2015) was used to evaluate the proportions of 22 tumor-infiltrating lymphocyte subsets in tumor samples before the estimation of the relative abundance of immune cell infiltration in patients with different ERBB4 statuses. The number of permutations was set to 1000, and the threshold of the criterion for successful computation of a sample was *p*-value 0.05.

### Statistical analysis

Statistical analyses were performed via the R software 3.6.3 and GraphPad Prism 7.0 software. Student’s t tests and one-way ANOVA test were conducted to determine the statistical significance. It has been shown that log-rank test was biased for two groups showing large differences in the number of samples. Therefore, Exact Log-rank Test (ExaLT) was performed to validated results of log-rank test (Vandin et al. 2015). All significant comparisons were considered a two-tailed *p*-value < 0.05.

## Results

### The role of ERBB4 and TP53 mutation in the prognosis of NSCLC patients analyzed by cBioPortal

**Fig. 1:**
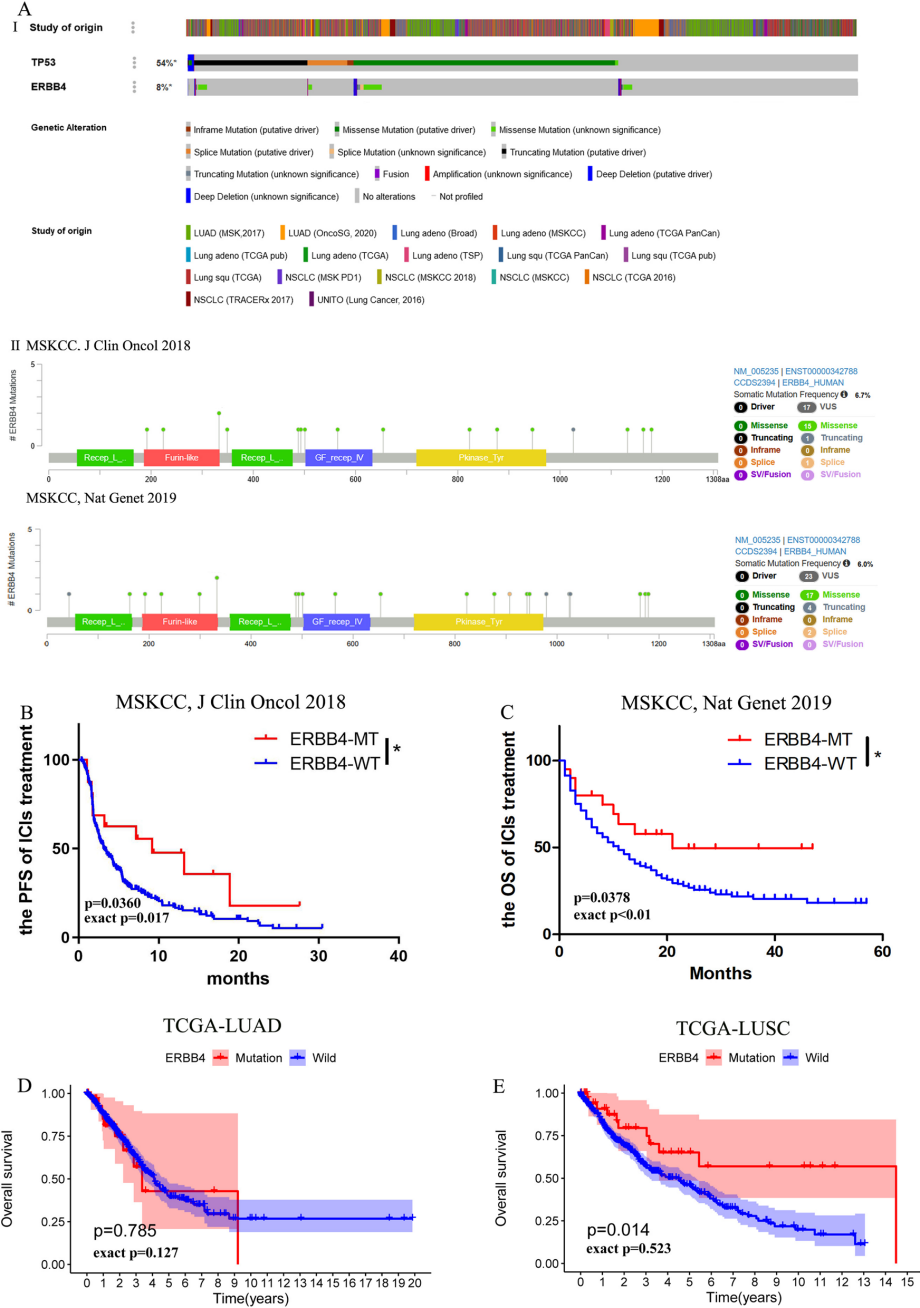
ERBB4 mutation are tightly associated with the prognosis of NSCLC. **AI** The prevalence of ERBB4 and TP53 mutations in NSCLC patients based on the cBioPortal for Cancer Genomics; **AII** The whole protein and specific changes of ERBB4 in the cohorts. **B** The relationship between ERBB4 mutation status and progress free survival (PFS) of NSCLC patients treated by ICIs; **C** The relationship between ERBB4 mutation status and overall survival (OS) of NSCLC patients treated by ICIs. **D** The relationship between ERBB4 mutation status and OS of LUAD patients treated by non-ICIs from the TCGA cohort. **E** The relationship between ERBB4 mutation status and OS of LUSC patients treated by non-ICIs from the TCGA cohort

Mutation frequencies of TP53 and ERBB4 were 54% and 8% in NSCLC, respectively (Fig. 1AI). In both cohorts, the mutual exclusive analysis in cBioPortal has indicated that ERBB4 does show co-occurencing mutations with TP53 (*p* < 0.01). Moreover, the TP53 mutation was found in all ERBB4-mutated patients of the both cohorts. According to the protein structure of ERBB4, the most common type of mutations has been missense in both cohorts (Fig. 1AII). The relationship between the survival of NSCLC patients and ERBB4 mutation status was further analyzed. According to the ERBB4 gene mutation status, advanced NSCLC patients treated with ICIs were divided into ERBB4-MT(16/240) and ERBB4-WT(224/240) groups, and the KM analysis was performed. The PFS and OS time period of the ERBB4-MT NSCLC patients (21/350) treated with ICIs was longer than that of the ERBB4-WT patients (329/350) (the median PFS: 9.2 vs 3.17 months, *p* = 0.0360; the median OS: 21 vs 11 months, *p* = 0.0378, Fig. 1B, C). Then, ExaLT was performed to validated results of log-rank test (PFS: exact *p* = 0.017; OS: exact *p* < 0.01). In addition, the somatic mutation and survival data of both TCGA-LUAD (mutated patients: 32/468) and TCGA-LUSC (mutated patients: 39/444) was downloaded from the Genomic Data Commons. According to the research herein, the ERBB4 gene mutation could not predict prognosis in the TCGA-LUSC or TCGA-LUAD patients who did not receive ICI treatment (LUSC: *p* = 0.014, exact *p* = 0.523; LUAD: *p* = 0.785, exact *p* = 0.127; Fig. 1D, E). Therefore, ERBB4-mutated patients exhibited a better prognosis than those with ERBB4 wild type treated with ICIs.

### The role of ERBB4 and TP53 mutation in outcome for ICIs treatment analyzed by cBioPortal

**Fig. 2:**
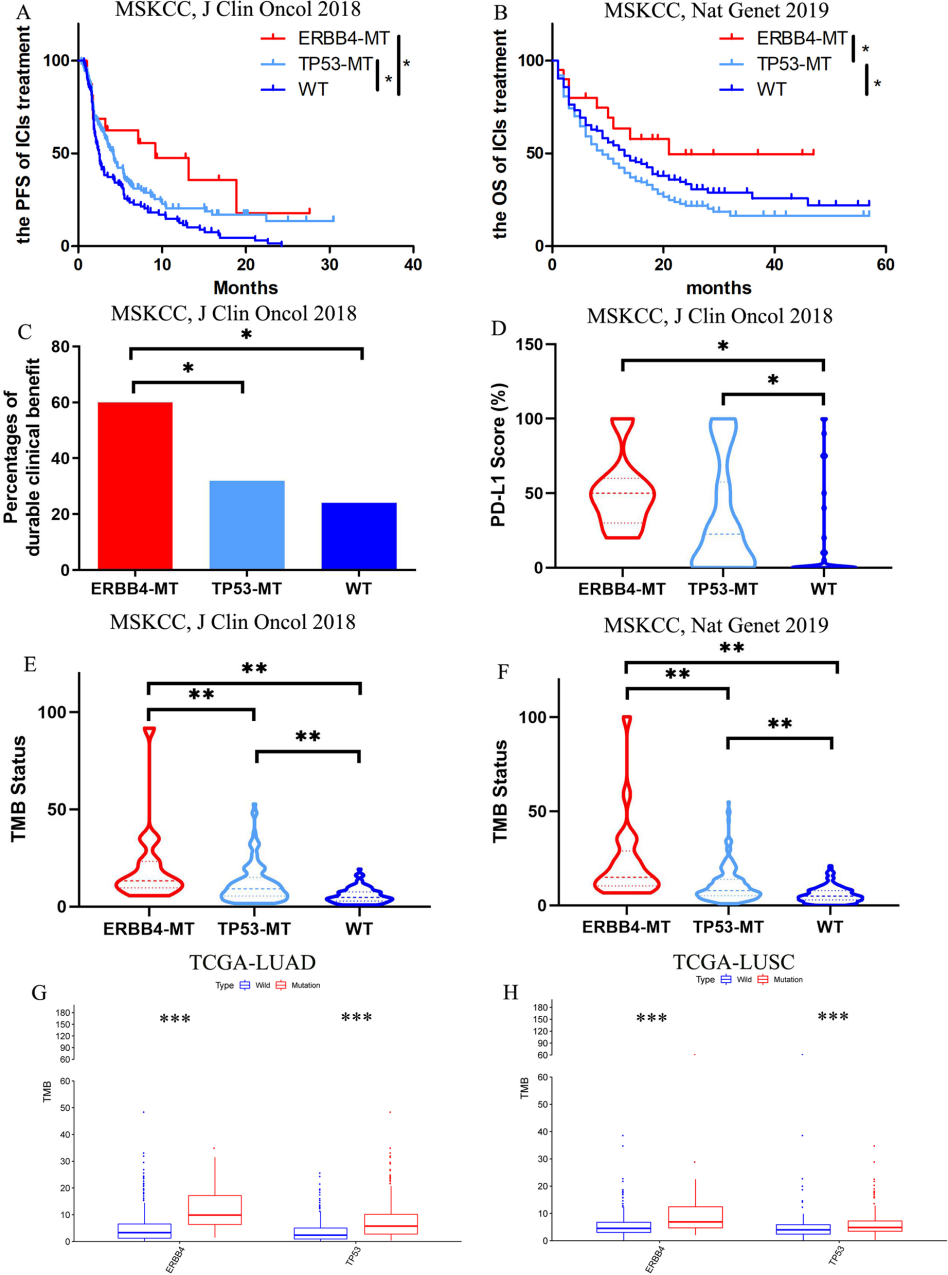
ERBB4 and TP53 co-mutation predict an improved prognosis of NSCLC with ICIs treatment. **A** The relationship between ERBB4 and TP53 co-mutation and PFS of NSCLC patients treated by ICIs; **B** The relationship between ERBB4 and TP53 co-mutation and OS of NSCLC patients treated by ICIs; **C** The mutation status of ERBB4 and TP53 is associated with the durable clinical benefit (DCB) in cBioPortal for Cancer Genomics; **D** The mutation status of ERBB4 and TP53 is associated with the PD-L1 expression in cBioPortal for Cancer Genomics; **E** The mutation status of ERBB4 and TP53 is associated with the tumor mutational burden (TMB) level in cohort [MSKCC, J Clin Oncol 2018] from cBioPortal for Cancer Genomics; **F** The mutation status of ERBB4 and TP53 is associated with the TMB level in cohort [TMB and Immunotherapy (MSKCC, Nat Genet 2019)] from cBioPortal for Cancer Genomics; **G** The mutation status of ERBB4 and TP53 is associated with the TMB level in TCGA-LUAD cohort; **H** The mutation status of ERBB4 and TP53 is associated with the TMB level in TCGA-LUSC cohort

Firstly, due to the TP53 mutation found in all ERBB4-mutated patients of the both cohorts, patients with TP53 mutation were divided into ERBB4-MT group (ERBB4 and TP53 comutation), TP53-MT group (TP53 mutation with ERBB4-wildtype), and WT group (without TP53 or ERBB4 mutation). The analysis of the datasets from the cBioPortal showed that the ERBB4 and TP53 comutation was associated with an improved sensitivity to immunotherapy. Although the difference of PFS between ERBB4-MT and TP53-MT was not significant (*p* = 0.135, Fig. 2A), patients harboring ERBB4 mutation were benefited more from ICIs treatment with a longer OS time period than those in the TP53-MT group (21 months vs 8 months, *p* = 0.021, Fig. 2B). As depicted in Fig. 2C, the percentage of DCB was higher in the ERBB4-MT group than that in the TP53-MT group and WT group (60% vs 31.9% vs 24%, ERBB4-MT vs TP53-MT group: *p* = 0.035; ERBB4-MT vs WT group: *p* = 0.021).

### The role of ERBB4 and TP53 mutation in the TMB level and PD-L1 expression analyzed by cBioPortal and TCGA

In addition, the mutation status of ERBB4 and TP53 was closely associated with the TMB level and PD-L1 expression (Fig. 2D–H). The ERBB4 and TP53 comutation was related to a higher PD-L1 score (proportion of PD-L1 positive cells) (ERBB4-WT VS MT group: *p* = 0.022, Fig. 2D) of advanced NSCLC patients. However, there was no significant difference of PD-L1 expression between the ERBB4-WT group and the TP53-WT group (*p* = 0.609). In both cohorts, a higher TMB value was confirmed in the ERBB4-MT group, compared to the figures for the TP53-MT and WT groups ([MSKCC, J Clin Oncol 2018]: TP53-MT group: *p* = 0.002, WT group: *p* < 0.001; [MSKCC, Nat Genet 2019]: TP53-MT group: *p* < 0.001, WT group: *p* < 0.001, Fig. 2E, F). Moreover, the ERBB4 and TP53 mutation was associated with a higher TMB value in both TCGA-LUAD and -LUSC cohorts, respectively (*p* < 0.001, Fig. 2G, H).

### Construction of nomogram for the prognosis of immunotherapy

**Fig. 3:**
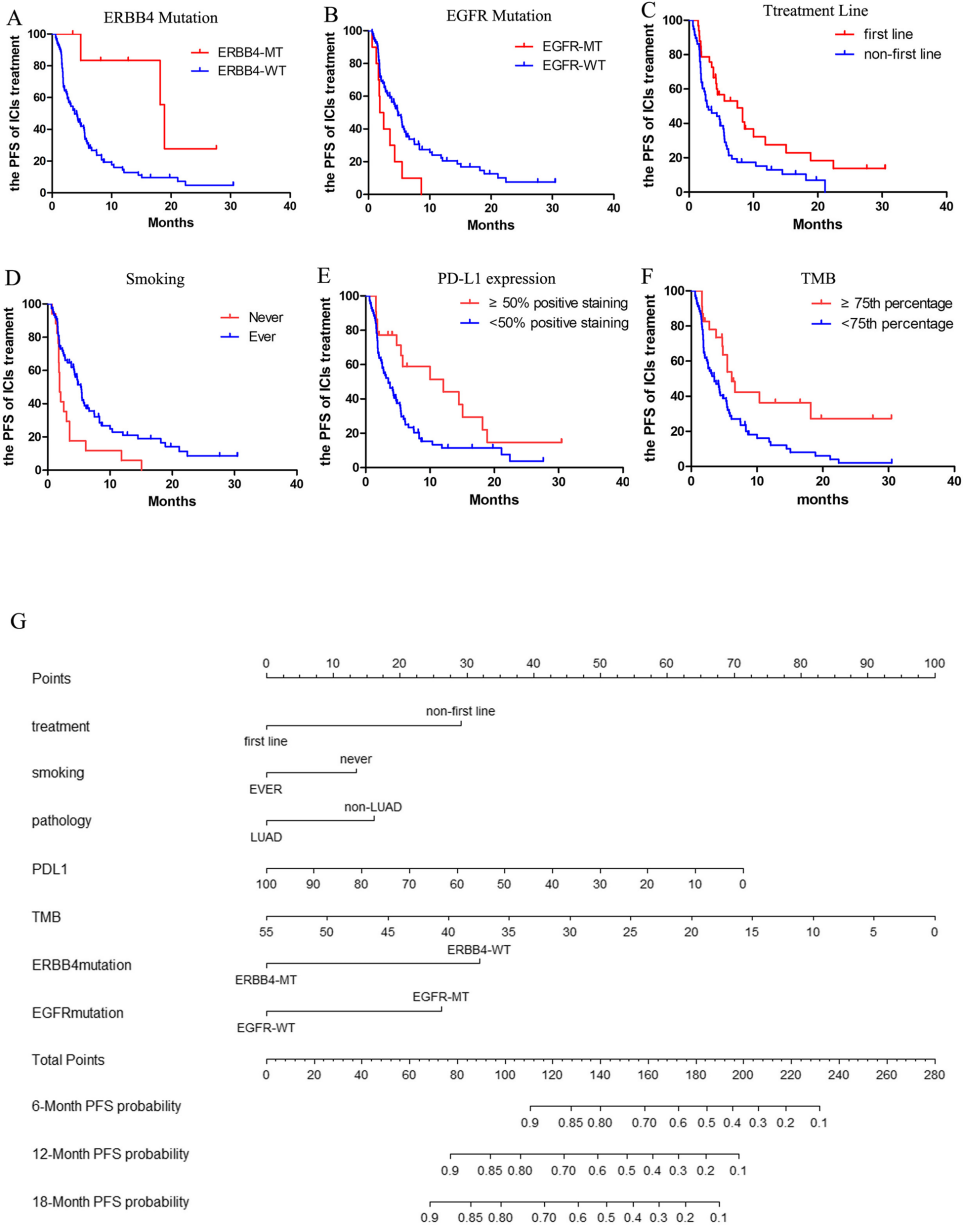

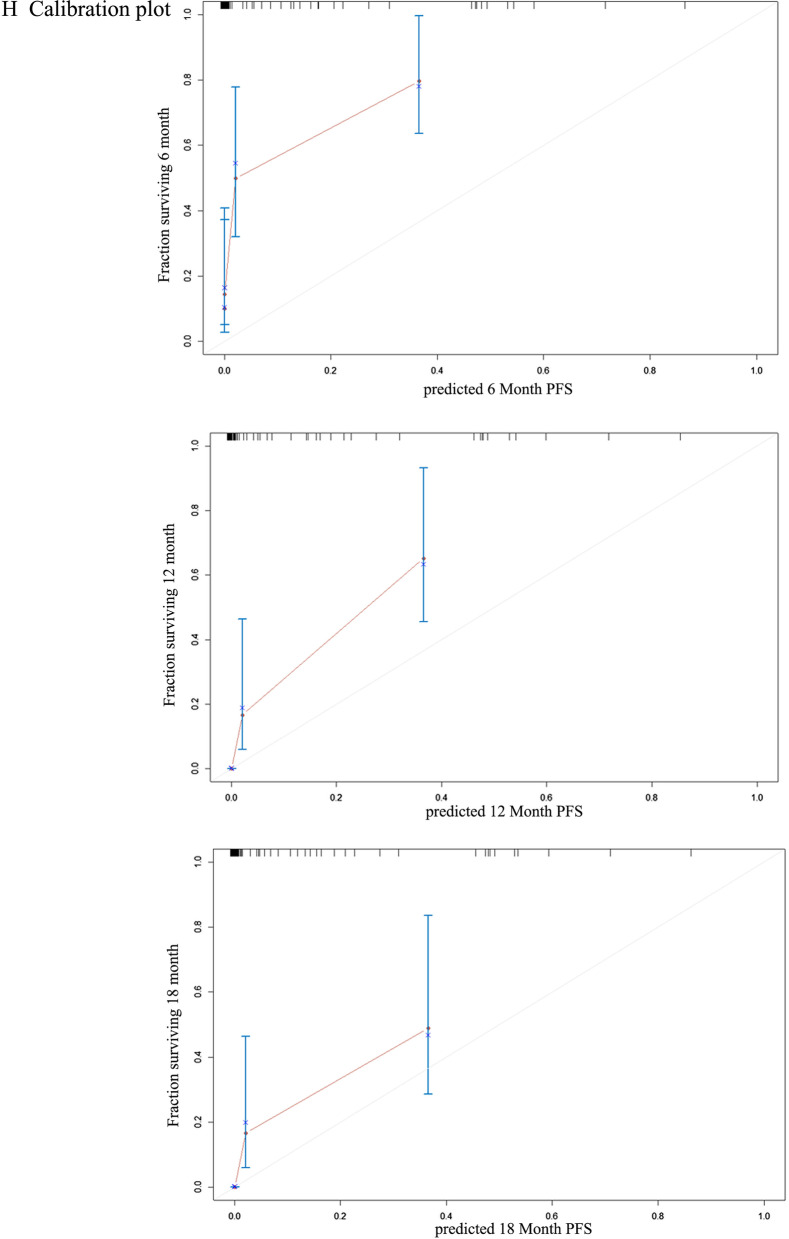
Novel nomogram to predict the prognosis of NSCLC patients with ICIs treatment. **A** The survival curves for patients with ICIs treatment based on the ERBB4 mutational status; **B** The survival curves for patients with ICIs treatment based on the EGFR mutational status; **C** The survival curves for patients with ICIs treatment based on PD-L1 expression; **D** The survival curves for patients with ICIs treatment based on the tumor mutational burden (TMB); **E** The survival curves for patients with ICIs treatment based on treatment lines; **F** The survival curves for patients with ICIs treatment based on smoking history; **G** The novel nomogram based on these variables to predict the prognosis of ICI treatment; **H** The calibration plot for the nomogram

The nomogram to predict the PFS of 83 NSCLC patients of the selected cohort was based on the integrated information of clinicopathologic features, targeted sequencing, and PD-L1 expression. Firstly, the univariate analyses were adopted in the identification of the variables for nomogram construction. Multiple variables were proved to be significantly linked to the PFS of NSCLC patients with ICI treatment, including ERBB4 mutation (*p* = 0.0079), EGFR mutation (*p* = 0.0152), smoking (*p* = 0.0040), treatment lines (*p* = 0.0097), TMB (*p* = 0.0059), and PD-L1 expression (*p* = 0.0113) (Fig. 3A–F). Moreover, the univariate analyses indicated that survival benefits from immunotherapy could be derived by advanced NSCLC patients with ERBB4 mutation, ever smoking, first-line ICIs administration, elevated expression of PD-L1 (≥ 50% percentage), or a high TMB score (≥ 75th percentage). However, the EGFR mutation was possible to predict the poor prognosis of NSCLC treated by ICIs. A systematic nomogram was formulated based on these variables (Fig. 3G), helping clinical physicians easily obtain a point of each variable and evaluate the total point as the sum of all variable points. Therefore, the efficacy of ICIs therapy for advanced NSCLC patients could be assessed in advance. The good accuracy of nomogram was demonstrated in estimating the PFS of advanced NSCLC patients with ICIs therapy through a bootstrap-corrected C index of 0.75 (95% CI 0.72 to 0.78). Calibration plots graphically showed the great predictive performance in Fig. 3H. As such, the ERBB4 mutation could be regarded as a novel prognostic biomarker for ICIs treatment, featuring a connection with factors that closely associated with the efficacy of immunotherapy.

### The role of ERBB4 mutation in tumor-infiltrating immune cell modulation and enrichment pathway analysis of ERBB4 mutation

**Fig. 4:**
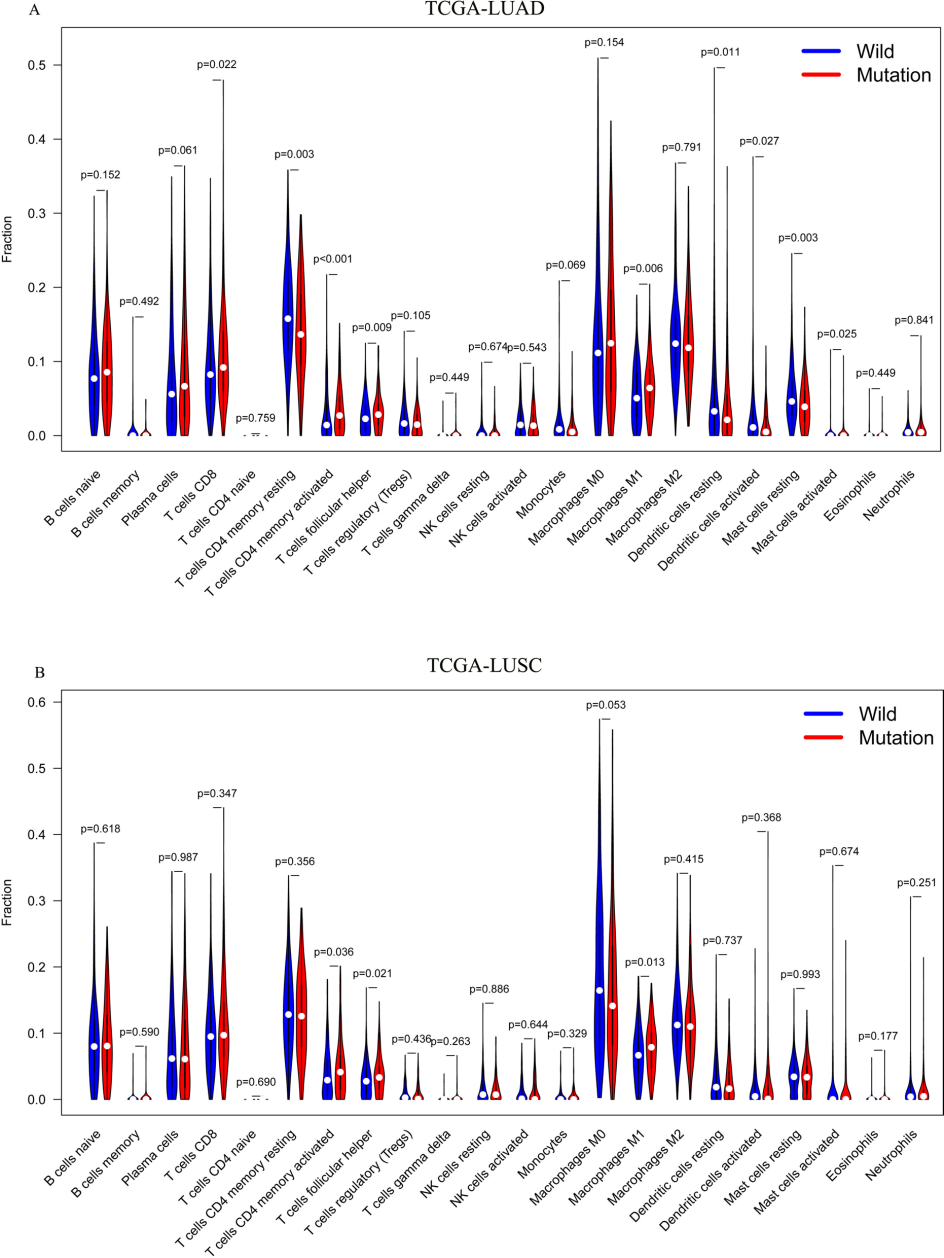
ERBB4 mutation is correlated with tumor-infiltrating immune cells. **A** Violin plot displays the differentially infiltrated immune cells between ERBB4-mutant groups and ERBB4-wild group in LUAD-TCGA cohort. **B** Violin plot displays the differentially infiltrated immune cells between ERBB4-mutant groups and ERBB4-wild group in LUSC-TCGA cohort

The relationship between ERBB4 mutation and tumor-infiltrating immune cells was analyzed with the help of CIBERSORT algorithm. As shown in Fig. 4A, CD8 T cells, activated memory CD4 T cells, follicular helper T cells, and M1 macrophages were more enriched in ERBB4 mutant LUAD group, while memory resting CD4 T cells, dendritic cells, and Mast cells were enriched in wild-type LUAD group. As for LUSC group, activated memory CD4 T cells, follicular helper T cells, and M1 macrophages were more abundant in ERBB4 mutant group (Fig. 4B). Therefore, it was possible for the deficiency of ERBB4 to activate the antigen presentation process and cellular immunity, leading to the change in the sensitivity to immunotherapy for advanced NSCLC patients.

**Fig. 5:**
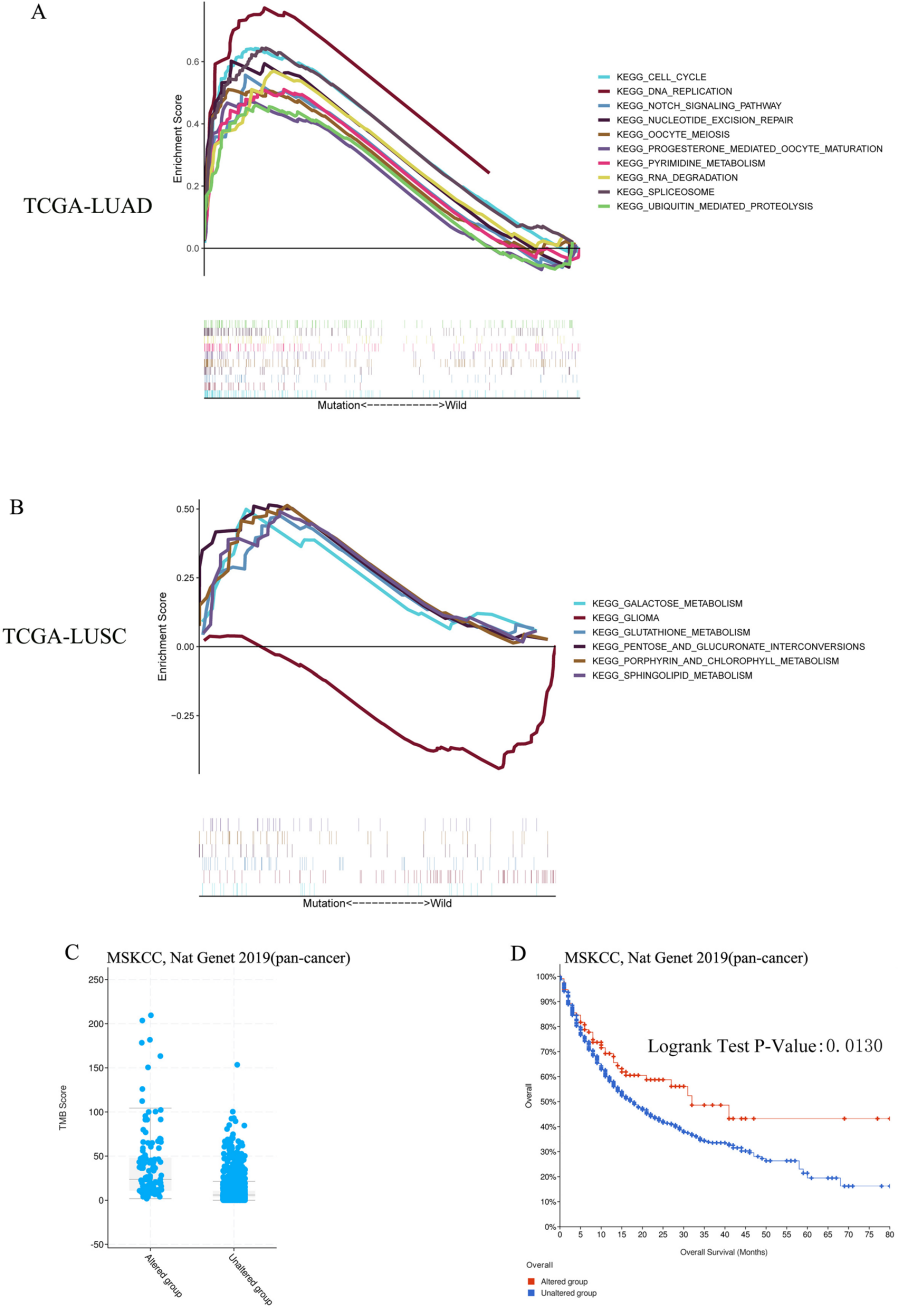
Significantly enriched pathways associated with ERBB4 mutation in NSCLC and prognostic value of ERBB4 in pan-cancer. **A** Significantly enriched pathways associated with ERBB4 mutation in LUAD-TCGA cohort. **B** Significantly enriched pathways associated with ERBB4 mutation in LUSC-TCGA cohort. **C** The mutation status of ERBB4 is associated with the TMB level in pan-cancer. **D** The survival curves based on the mutational status of ERBB4 in pan-cancer

The GSEA analysis performed with TCGA-LUAD revealed that Cell Cycle, Oocyte Meiosis, Spliceosome, RNA Degradation, Nucleotide Excisio, Repair DNA Replication, Notch Signaling Pathway Progesterone Mediated Oocyte Maturation, Pyrimidine Metabolism, and Ubiquitin Mediated Proteolysis pathway were significantly enriched in samples with ERBB4 mutation (Fig. 5A). As for TCGA-LUSC samples, Porphyrin and Chlorophyll Metabolism, Galactose Metabolism, Sphingolipid Metabolism Pentose and Glucuronate Interconversions, and Glutathione Metabolism Pathway were significantly abundant in samples with ERBB4 mutation (Fig. 5B). However, Glioma pathway was enriched in samples with wild type.

### Prognostic value of ERBB4 in pan-cancer

The prognostic value of ERBB4 was an independent external validation in the cohort [TMB and Immunotherapy (MSKCC, Nat Genet 2019)] (Samstein et al. 2019) in cBioPortal of Cancer Genomics, according to the analysis herein. The mutation of ERBB4 led to a higher TMB level in various cancers (*p* < 0.0001) (Fig. 5C). The Kaplan–Meier survival analysis indicated that the mutation status of ERBB4 was associated with the prognosis of cancer patients receiving ICIs treatment (*p* = 0.0130) (Fig. 5D).

## Discussion

It is worth noting that the PD-L1 expression or TMB value did not show satisfying efficiency in the selection of patients who might get benefits from immunotherapy (Sun et al. 2020; Wang et al. 2021a, b). Recently, some specific gene mutations have been disclosed to have the intimate relationship with the efficacy of ICIs treatment. Although TP53 has seen the prevalence of alteration in NSCLC, the outcomes of its mutations have not been the same among patients with ICIs treatment all the time (Wang et al. 2021a, b). For the patients receiving atezolizumab or docetaxel, the better survival benefit of ICIs treatment could be found in PD-L1 positive patients with the TP53 mutation (Wang et al. 2021a, b). Moreover, the TP53 co-mutation with EGFR/STK11/KRAS/ATM has been proved to have the predictive value for the outcome of ICIs in NSCLC (Chen et al. 2019; Dong et al. 2017; Biton et al. 2018; Skoulidis et al. 2015). Therefore, these gene mutations might be beneficial to predicting the prognosis of ICIs treatment for advanced patients with mutated-TP53. In addition, Wang et al. (2021a, b) indicated that the mutation of FGFR4 might serve as a novel biomarker in modulating the TIME, correlated with the prognosis of NSCLC patients. Data from the Cancer Genome Atlas showed that the loss of ERBB4 gene copy numbers was found in different cancer types, including esophageal, lung, bladder and cervical carcinoma (Segers et al. 2020). A similar pattern could be proven from mRNA expression analyses of a large fraction of tumor cell lines. RNA sequencing data from the Cancer Cell Line Encyclopedia (CCLE) has indicated that the mRNA expression of ERBB4 is down-regulated in different tumor-derived cell lines (Segers et al. 2020). Therefore, the functional deficiencies of ERBB4 might promote tumor growth in various types of cancers, associating with the poor prognosis of cancer patients (Long et al. 2003; Jones et al. 1999). The main signaling pathways related to ERBB4 are the Ras–MAPK–ERK and PI3K–Akt pathways, moderating the cell cycle cessation and differentiation (Iwakura and Nawa 2013; Telesco et al. 2013). Naresh et al. (2006) have implied that the somatic mutations of ERBB4 in cancer suppress both pathways and give rise to cell proliferation rather than differentiation (Long et al. 2003; Jones et al. 1999). Consequently, the tumor suppressor-like function of ERBB4 is strongly supported. Studies on the role of ERBB4 in the immune system are relatively new. It has been reported that the activation of ERBB4 receptors may lead to macrophage apoptosis in a mouse model of colitis (Schumacher et al. 2017). Moreover, in another mouse model of cardiac and skin fibrosis, the activation of ERBB4 receptors on macrophages makes contributions to the attenuation of inflammation and fibrosis (Vermeulen et al. 2017; De Keulenaer et al. 2019). Based on these former studies, the research has been conducted on the further investigation into the predictive value of ERBB4 mutations seen by advanced NSCLC patients with ICIs treatment.

In the study hereof, ERBB4 did show co-occurencing mutations with TP53. Moreover, ERBB4 and TP53 comutation was associated with clinical benefits and the survival improvement, compared with the only mutation of TP53. Though their PFS time did not show significant differences between ERBB4-TP53 comutation and only TP53 mutation, the OS of patients with ERBB4 and TP53 comutation was proven to be prolonged through ICIs therapy. Additionally, the comutation of TP53 and ERBB4 was closely related to other predictive biomarkers of ICIs therapy, such as the expression of PD-L1, TMB value, and TIICs. NSCLC patients harboring ERBB4 and TP53 comutations might boost TMB and PD-L1 expression. What’s more, ERBB4 and TP53 deficiencies could moderate the infiltrating immune cells and augment tumor immunogenicity by activating the process of antigen presentation and anticancer cellular immunity in patients with NSCLC. As a result, ERBB4 mutation might be closely associated with an additional clinical benefit for patients with mutated TP53. The GSEA enrichment analysis showed that these ERBB4-MT groups were mainly associated with Cell Cycle, Oocyte Meiosis, Spliceosome, and RNA Degradation pathways in LUAD and Porphyrin and Chlorophyll Metabolism, Galactose Metabolism, Sphingolipid Metabolism Pentose pathways in LUSC, respectively. According to the results of Cox regression, a novel nomogram was formulated based on the mutation statues of EGFR and ERBB4, PD-L1 expression, TMB level, and other clinicopathological features of advanced NSCLC patients with ICIs therapy. This might assist patients and physicians to estimate the clinical benefits of ICIs therapy and determine the appropriate therapeutic plan and follow-ups before the treatment for patients with NSCLC.

The existing reports have indicated that further exploration should be carried out on the mechanism of ERBB4 witnessed by advanced NSCLC patients with ICIs treatment. The research herein has taken the lead in the investigation into the possibility of ERBB4 mutation serving as a novel biomarker in modulating the TIME and correlating with the prognosis of NSCLC patients with ICIs therapy. In addition, the tendency between ERBB4 and TP53 mutation, together with their role in PFS and OS of NSCLC patients with ICIs therapy, has been clearly clarified. At the same time, the functional deficiencies of ERBB4 can make contributions to the poor prognosis of NSCLC patients. The mutation of ERBB4 is closely associated with the change of cancer phenotype and has shown the predictive value in mutated-TP53 patients with ICIs therapy. Nevertheless, several limitations of the study herein have been presented as follows. Firstly, the further analysis of ERBB4 function in NSCLC with ICIs therapy is needed because of small clinical sample size in this research. In addition, the molecular mechanism underlying the association of ERBB4 mutation with the modulation of the TIME and a higher TMB and PD-L1 expression in NSCLC are still unclear. Last but not least, the full implications of ERBB4 mutation remain elusive, a field where further studies are required.

## Conclusion

There is a chance for ERBB4 to serve as a novel biomarker for advanced NSCLC treated by ICIs. The mutation status of ERBB4 and TP53 are tightly linked to the prognosis for ICIs treatment, PD-L1 expression TMB value, and TIICs. The systematic nomogram has been formulated based on these biomarkers to assess the efficacy of ICIs therapy for advanced NSCLC patients.

## Data Availability

All data generated during this study are included herein. The datasets generated in the current study are available in the cBioportal of Cancer Genomics (Cerami et al. 2012; Gao et al. 2013) (http://www.cbioportal.org/).

## Abbreviations

ERBB4: Erb-b2 receptor tyrosine kinase 4
NSCLC: Non-small cell lung cancer
MMR: Mismatch repair
MSI-H: Microsatellite instability-high
PD-L1: Programmed death ligand 1
TMB: Tumor mutational burden
ICIs: Immune checkpoint inhibitors
TIICs: Tumor-infiltrating immune cells
TCGA: The Cancer Genome Atlas
PFS: Progress free survival
OS: Overall survival
GSEA: Gene set enrichment analysis
NGS: Next-generation sequencing
ExaLT: Exact Log-rank Test
